# Contemporary factors affecting serum 25-hydroxyvitamin D concentrations in Chinese children aged 2–6 years

**DOI:** 10.1017/S1368980024001320

**Published:** 2025-06-04

**Authors:** Qian Chen, Ting Yang, Yongfang Liu, Jie Chen, Qian Cheng, Tingyu Li

**Affiliations:** 1 Department of Child Health Care, Children’s Hospital of Chongqing Medical University, National Clinical Research Center for Child Health and Disorders, Ministry of Education Key Laboratory of Child Development and Disorders, Chongqing Key Laboratory of Child Health and Nutrition, Chongqing 400014, China; 2 Department of Nutrition, Children’s Hospital of Chongqing Medical University, Chongqing, China

**Keywords:** Vitamin D, Children, Deficiency, Insufficiency, China

## Abstract

**Objective:**

We investigated vitamin D (VitD) nutritional status in children aged 2–6 years to provide a basis for prevention and intervention strategies for VitD deficiency (VitDD) in Chinese children.

**Design:**

From November 2018 to September 2019, a total of 2192 healthy children aged 2–6 years were enrolled. The serum 25-hydroxyvitamin D (25(OH)D) concentrations were measured by liquid chromatography tandem MS.

**Setting:**

Twelve jurisdictions in eight provinces and cities across northern and southern China were selected through stratified cluster sampling.

**Participants:**

2192 children aged 2–6 years were enrolled.

**Results:**

(1) A serum 25(OH)D concentration of 23·87 (sd 8·24) ng/ml, a VitDS rate of 65·2 %, an insufficiency rate of 29·6 % and a deficiency rate of 5·2 % were noted. (2) Age (OR = 2·22, 95 % CI 1·86, 2·64) and spring (OR = 1·35, 95 % CI 0·91, 2·01) are risk factors for VitDD and VitDI. The male (OR = 0·68, 95 % CI 0·52, 0·90), the temperature (OR = 0·89, 95 % CI 0·86, 0·93), summer (OR = 0·25, 95 % CI 0·09, 0·68), autumn (OR = 0·26, 95 % CI 0·09, 0·74) the intake of VitD supplements (OR = 0·08, 95 % CI 0·03, 0·28), the intake frequency of dairy products (OR = 0·86, 95 % CI 0·78, 0·96) and egg products (OR = 0·83, 95 % CI 0·74, 0·93) are protective factors for VitDD and VitDI.

**Conclusion:**

VitDD in children aged 2–6 years is still prevalent in China, but the influencing factors of VitD nutrition have changed. Latitude is not the main factor in the 25(OH)D concentrations of children aged 2–6 years; temperature, intake of eggs and dairy products and sampling season have more obvious impacts.

Vitamin D (VitD) is a class of lipid-soluble vitamins. VitD not only plays a role in regulating Ca and phosphorus metabolism and bone health but also has a wider range of extraosseous biological effects^(1)^. It is associated with obesity, muscle, CVD, endocrine and metabolism, infections, and immune diseases^(1,2)^. VitD deficiency (VitDD) in children may also be associated with many diseases in adulthood. Therefore, VitD status in childhood has received more attention^(3)^. With the intensification of air pollution and the corresponding reduction in outdoor activities, VitDD has become increasingly common in humans, especially children, and VitD insufficiency and deficiency have become a public health problem in developed and developing countries^(4)^.

The metabolism and nutritional status of VitD in the human body are affected by various factors, such as race, region, sex and age^(3,5–10)^. China has a large inland area, a large span between north and south latitudes, and a large difference in altitude between east and west. There are significant differences in temperature between different regions. People in different regions receive different intensities of sunlight and have different dressing and outdoor activity habits, which directly affect the concentrations of 25-hydroxyvitamin D (25(OH)D) synthesis by the local residents. Even in the same province, the 25(OH)D concentration is still affected by different altitudes and light levels^(11)^. Therefore, it is very important to investigate the current nutritional status of VitD in China on a large scale, but the most recent national VitD survey in China was from 2010 to 2013^(12)^. It suggested that the VitDD rate of children aged 3–5 years in China was 8·9 %. In recent years, although there have been many studies on the nutritional status of VitD in China, they are all based on the physical examination data of children in local hospitals or healthcare centres^(3,4,13–16)^, which have a significant bias. The most recent multicentre survey of VitD status in the general population was conducted in 2013–2015, and only people aged 6–18 years in six cities were surveyed^(17)^. Since 2013, there has been no multicentre survey based on VitD status in preschool children. Due to the lack of large-scale population data, the ‘Expert Consensus on the Clinical Application of Vitamin A and Vitamin D in Chinese Children’ published in China in 2021 still used the data from the 2010–2013 survey^(18)^. A new population-based multicentre VitD nutrition survey in children is needed.

This study was a multicentre cross-sectional study. The VitD and dietary nutritional status of children aged 2–6 years in twelve regions of eight provinces and cities in China were sampled and analysed. The influencing factors of VitD nutritional status were analysed to understand the recent changes in VitD nutrition and its influencing factors in China and provide a basis for adjusting China’s VitD intervention policy.

## Objectives and methods

### Research subjects and sampling strategy

This study was a multicentre cross-sectional survey. The sampling period was November 2018 to September 2019. The methods were approved by the Ethics Committee.

The study sample size was calculated using the following formula based on relevant research reports on VitDD: 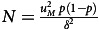 . The prevalence of VitDD (< 12 ng/ml) has been calculated at 8·9 %(12). Given a probability of type I errors less than 0·05, the allowable errors between the sample prevalence and the overall prevalence were less than 5 %. This study needed a sample size of 125 based on the following calculation:

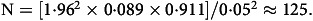




Assuming a loss-to-follow-up rate of 10 %, the minimum sample size was 138. Therefore, we selected 200 children from each sampling site.

**Fig. 1:**
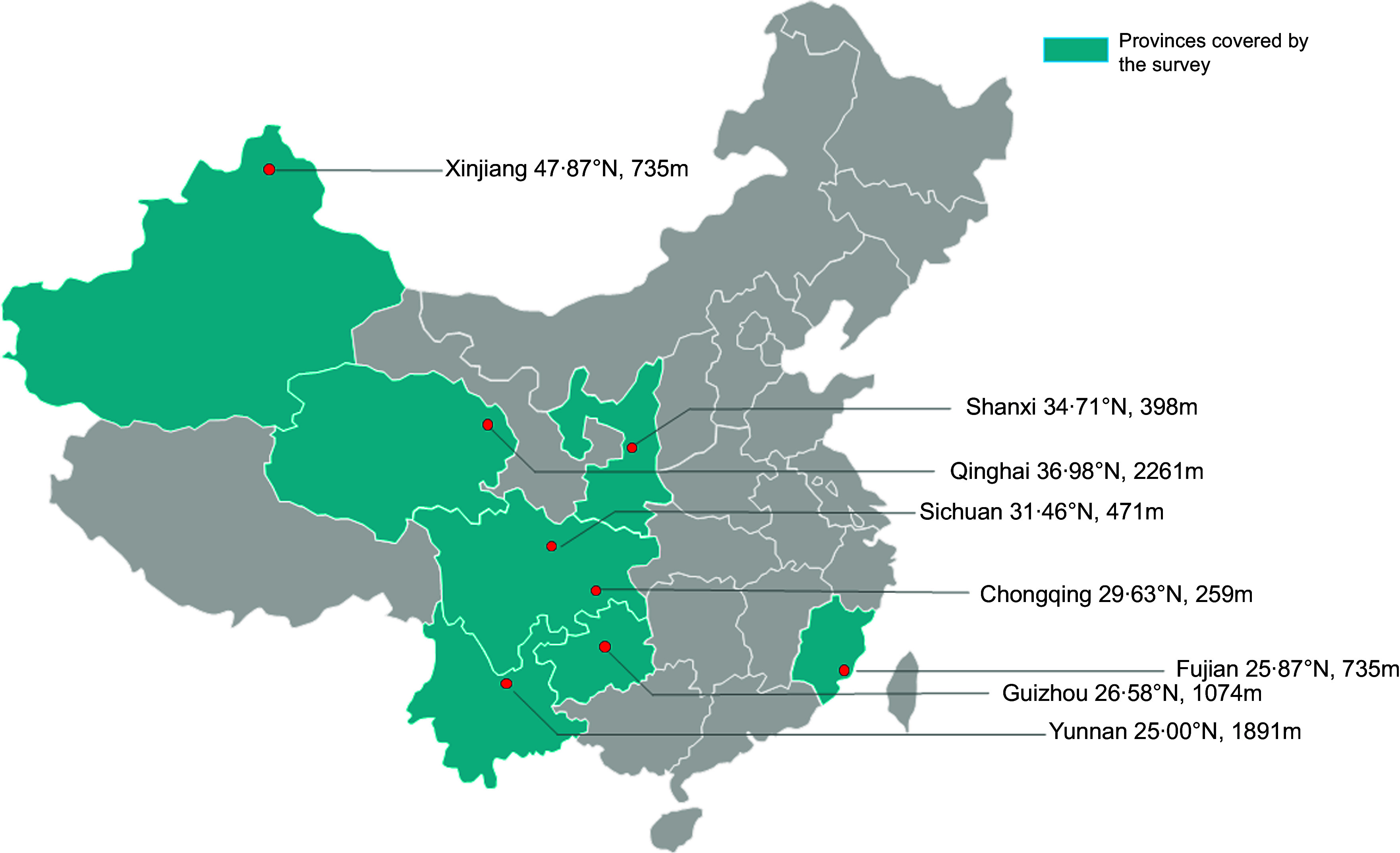
Regions covered by this study

Sampling sites were chosen from provinces/municipalities, and the sampled provinces ran from the north to the south of China. The northernmost region was the Altay Prefecture of Xinjiang, and the southernmost region was Yunnan. Cities with different geographical characteristics were selected near the same latitude: the coastal city of Fuzhou in Fujian Province and the inland cities of Guiyang and Kunming. From each area, twelve sampling points were randomly selected by computer-generated random numbers. The final selected regions were Chongqing (Bishan, Liangjiang New District, Changshou, Qianjiang and Wanzhou), Guizhou (Guiyang), Yunnan (Kunming), Sichuan (Mianyang), Qinghai (Xining), Shaanxi (Xianyang), Fujian (Fuzhou) and Xinjiang (Altay) (Fig. 1).

A cross-sectional study was conducted to enrol healthy children aged 2–6 years who lived in the zones mentioned above. Children of appropriate ages were randomly selected from randomly selected kindergartens. Hospitals in Wanzhou, Mianyang, Qianjiang and Kunming also enrolled eligible participants from their respective societies. The specific situation of this queue is described in detail in Chen Qian’s article published in 2021^(19)^. The inclusion and exclusion criteria are also the same.

Four millilitres of postprandial venous blood were collected from the children in the morning, the serum was segregated by centrifugation and a third-party company quantified the 25(OH)D concentration. The 25(OH)D concentration, including 25(OH)D_3_ and 25(OH)D_2_, was determined with liquid-phase tandem MS (AB SCIEX Framingham). A triple-quad mass spectrometer equipped with an Agilent Zorbax Eclipse XDB C8 guard column was used in the testing. The standard reference material VitD in human serum are ‘740 217’ and ‘739 650’ from the company of Sigma. The limit of quantitation and limit of detection were 1·2 ng/ml and 0·4 ng/ml, respectively. The intraday and interday precision were 3·27 % and 5·97 %, respectively. The method is accredited by the Vitamin D External Quality Assessment Scheme. The sum of 25(OH)D3 and 25(OH)D2 equals the total plasma 25(OH)D concentrations among the participants.

### Evaluation criteria

Due to the limited understanding of the physiological functions of VitD, the definition of VitD serum status has been controversial. Currently, the serum VitD concentration cut-off point published by the Institute of Medicine in 2011 is the most widely accepted. The ‘Expert Consensus on the Clinical Application of Vitamin A and Vitamin D in Chinese Children’ published in 2021 also recommends the use of this threshold^(18)^.

- VitD sufficiency (VitDS): serum 25-hydroxy-VitD (25(OH)D) ≥ 50 nmol/l (20 ng/ml),
- VitD insufficiency (VitDI): serum 25(OH)D 30–50 nmol/l (12–20 ng/ml),
- VitD insufficiency (VitDI): serum 25(OH)D 30–50 nmol/l (12–20 ng/ml),
- VitD deficiency (VitDD): serum 25(OH)D < 30 nmol/l (12 ng/ml)^(21)^
- VitD inadequate: VitDI + VitDD: serum 25(OH)D < 50 nmol/l (20 ng/ml)
  Units of measurement used internationally to quantify VitD include^(10)^:
- Doses: 1 microgram (µg) = 2·5 nanomoles (nmol) = 40 international units (IU)
- Concentrations: 1 ng/ml = 1 µg/l = 2·5 nmol/l

### Natural environment data

Data on the children’s area of residence, including the elevation and latitude data of the various regions, came from the ‘Load Code for the Design of Building Structures (2006 Edition) (GB500092012) [S]’^(22)^. The annual mean temperature data came from the National Earth System Science Data Sharing Service Platform.

### Data processing and statistical methods

A predefined testing centre in each area carried out all the tests, and the data were double-entered into Excel 2007 software (Microsoft, Redmond, USA). Continuous data are expressed as the mean (sd), and Student’s *t* test was used to compare independent samples. To compare differences between three or more groups, we used one-way ANOVA. The *χ* ^2^ test was used to compare proportions between groups. In the logistic regression analysis, VitD nutritional status was the dependent variable (1 = VitD inadequate: VitDI + VitDD, 0 = VitDS). The final correction factors included in the logistic regression model were weight-for-age Z-score (WAZ), height-for-age Z-score (HAZ) and BMI Z-score (BMZ). The independent variables of each grade were assigned as follows: sex (1 = male, 0 = female), age, season (1 = spring, 2 = summer, 3 = autumn, 4 = winter), vitD intake (0 = without vitD supplementation, 1 = with vitD supplementation), and milk intake and egg intake (9 = eat every day, 8 = 3–5 times/week, 7 = 1–2 times/week, 6 = little, 5 = never).

The logistic regression model for evaluation was run after adjustment for the WAZ, HAZ and BMZ. A stepwise selection technique was applied to include variables with a significance level for entry > 0·10 and exclude those with a significance level to stay < 0·05. All statistical analyses were performed in SAS 9.4 software (SAS Institute). *P* < 0·05 was considered statistically significant.

## Results

### Overall demographic and vitamin D nutritional status of the sample

In this study, a total of 2192 subjects met the inclusion criteria. Their average age was 3·83 (sd 1·14) years. The boys numbered 1165, accounting for 53·1 %, and the sex composition ratio did not significantly differ from that expected. The average concentration of 25(OH)D in the total population was 23·87 (sd 8·24) ng/ml, 5·2 % of children had VitDD (25(OH)D < 12 ng/ml) and 29·6 % of children had VitD insufficiency (12 ng/ml ≤ 25(OH)D < 20 ng/ml). The 25(OH)D concentration in boys was higher than that in girls (24·3 (sd 8·33) ng/ml *v*. 22·66 (sd 7·98) ng/ml, *P* < 0·05), and the VitD sufficiency rate in boys was higher than that in girls (70·1 % *v*. 59·6 %, *P* < 0·05, Table 1). There were no significant differences in age, region of origin, blood collection season or dietary habits between males and females (Table 1).

**Table 1:** Basic demographic characteristics and dietary intake of participants (*n* 2192)

	Total			Male			Female			
	*n* 2192 (%)			*n* 1165 (%)			*n* 1027 (%)			
Variable		Mean	sd		Mean	sd		Mean	sd	*P* value
Age (years)		3·83	1·14		3·81	1·14		3·84	1·15	0·501
25(OH)D, ng/ml		23·87	8·24		24·93	8·33		22·66	7·98	< 0·000
Vitamin D nutritional status	*n*	%		*n*	%		*n*	%		
Sufficiency (≥ 20 ng/ml)	1429	65·2		817	70·1		612	59·6		< 0·000
Insufficiency (12–20 ng/ml)	649	29·6		305	26·2		344	33·5		< 0·000
Deficiency (< 12 ng/ml)	114	5·2		43	3·7		71	6·9		
Sampling site, *n* (%)										
Chongqing (5 primary urban districts and suburban districts)	827	37·7		422	36·2		405	39·4		0·190
Guizhou (Guiyang)	198	9·0		111	9·5		87	8·5		0·190
Yunnan (Kunming)	201	9·2		96	8·2		105	10·2		
Sichuan (Mianyang)	210	9·6		111	9·5		99	9·6		
Qinghai (Xining)	151	6·9		82	7·0		69	6·7		
Shaanxi (Xianyang)	224	10·2		121	10·4		103	10·0		
Fujian (Fuzhou)	181	8·3		111	9·5		70	6·8		
Xinjiang (Altay)	200	9·1		111	9·5		89	8·7		
Blood collection season, *n* (%)										
Spring (March to May)	1361	62·1		746	64·0		615	59·9		0·207
Summer (June to August)	252	11·5		132	11·3		120	11·7		0·207
Fall (September to November)	96	4·4		48	4·1		48	4·7		
Winter (December to February)	483	22·0		239	20·5		244	23·8		
Intake frequency ≥ 3 times/week		*n* 1833(%)								
Dairy products, *n* (%)	1326	72·3		721	54·4		605	45·6		0·101
Eggs, *n* (%)	1168	63·7		641	54·9		527	45·1		0·055
Aquatic products, *n* (%)	286	15·6		152	53·2		134	46·9		0·987
Meat, *n* (%)	1317	71·8		699	53·1		618	46·9		0·873
Physical measures		*n* 1437 (%)								
		Mean	sd		Mean	sd		Mean	sd	
HAZ		−0·10	1·58		−0·12	1·57		−0·06	1·61	0·537
WAZ		0·07	1·18		0·11	1·21		0·04	1·15	0·206
BMI Z-score (kg/m^2^)		0·21	1·12		0·29	1·20		0·12	1·01	0·003
	*n*	%		*n*	%		*n*	%		
Stunting	93	6·47		52	6·70		41	6·20		0·702
Underweight	44	3·06		27	3·48		17	2·57		0·320
Wasting	32	2·23		19	2·45		13	1·97		0·681
Overweight	51	2·58		22	2·84		15	2·27		0·681
Obese	14	0·97		9	1·16		5	0·76		0·681

25(OH)D, 25-hydroxyvitamin D; HAZ, height-for-age Z-score; WAZ, weight-for-age Z-score.

Continuous variables are expressed as the mean (sd), and categorical variables are expressed as the number of samples (%).

VDS, vitamin D sufficiency: 25(OH)D > 20 ng/ml, VDI, vitamin D insufficient: 12–20 ng/ml, VDD, vitamin D deficiency < 12 ng/ml. #Significant difference in prevalence of VDD, VDI and VDS (*P* < 0·05).

### Vitamin D nutritional status by age, season and region

**Fig. 2:**
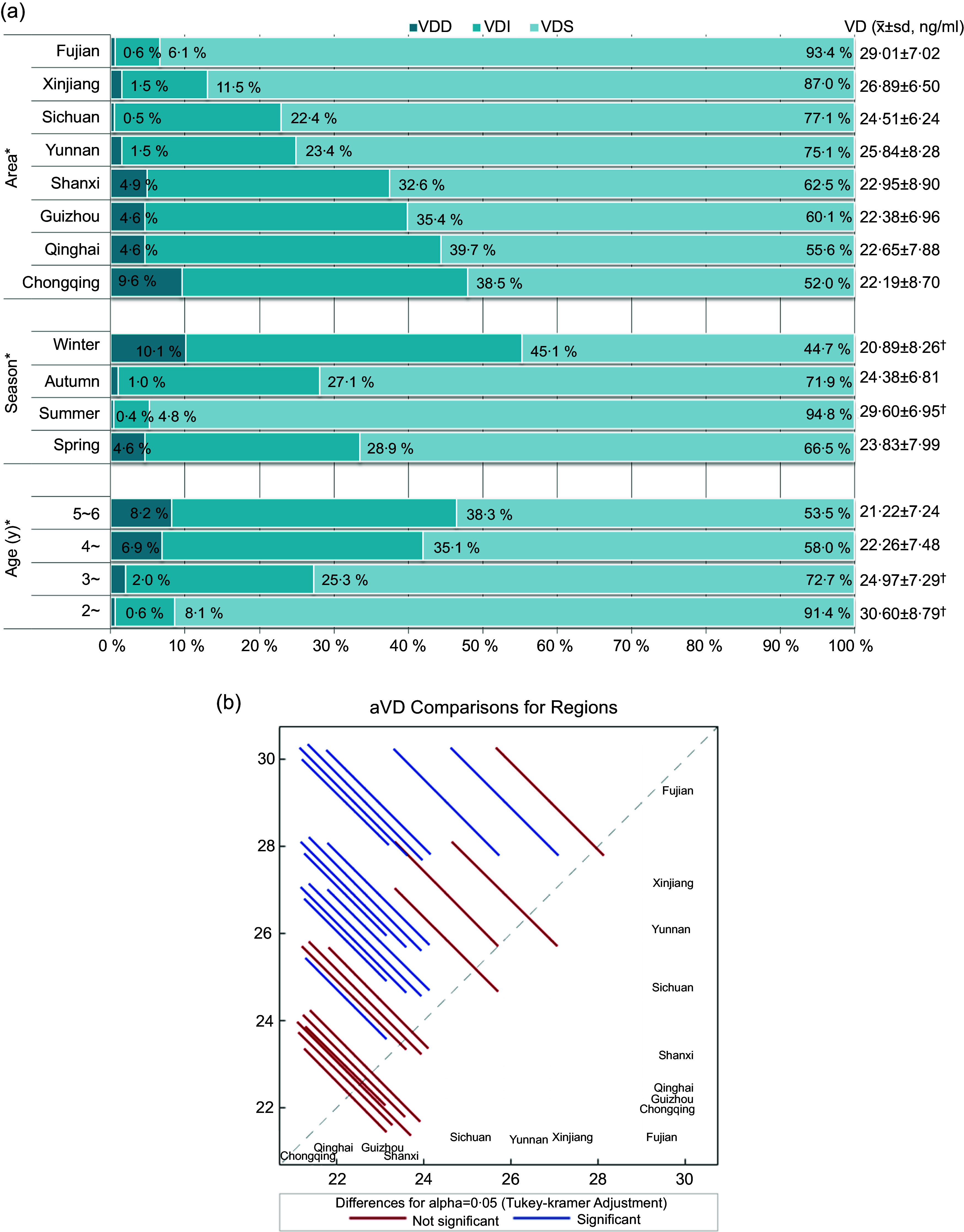
(a) Vitamin D nutritional status by age, season and region. VDS, vitamin D sufficiency: 25(OH)D > 20 ng/ml; VDI, vitamin D insufficiency: 12–20 ng/ml; VDD, vitamin D deficiency: < 12 ng/ml. * Significant difference in prevalence of VitDD, VitDI, and VitDS (*P* < 0·05). †Significant difference in VitD concentrations between groups (*P* < 0·05). (b) The serum 25(OH)D concentration in different regions. VitD, vitamin D; VitDD, VitD deficiency; VitDI insufficiency; VitD sufficiency; 25(OH)D, 25-hydroxyvitamin D.

When the children were grouped by age, the serum 25(OH)D concentration and VitD sufficiency rate of children aged 2–6 years gradually decreased with age (30·60 (sd 8·79) ng/ml, 91·4 %; 24·97 (sd 7·29) ng/ml, 72·7 %; 22·26 (sd 7·48) ng/ml, 58·0 %; and 21·22 (sd 7·24) ng/ml, 53·5 %). The 25(OH)D concentrations of the 2-year-old and 3-year-old groups were significantly different from those of the other groups (*P* < 0·05), and there was no significant difference between the 4-year-old and the 5–6-year-old groups (*P* > 0·05). With increasing age, the incidence of VitD deficiency and insufficiency gradually increased (*P* < 0·05) (Fig. 2(a)).

The serum 25(OH)D concentration varied with the season, and the prevalence of VitD deficiency and insufficiency showed the opposite trend (Fig. 2(a)). The 25(OH)D concentration was highest in summer (29·60 (sd 6·95) ng/ml, VitD sufficiency rate of 94·8 %), followed by 24·38 (sd 6·81) ng/ml in fall (VitD sufficiency rate of 71·9 %) and was lowest in winter (20·89 (sd 8·26) ng/ml, VitD sufficiency rate of 44·7 %) (*P* < 0·05).

Among the eight provinces studied, Fujian had the highest 25(OH)D concentration and VitD sufficiency rate (29·01 (sd 7·02) ng/ml, 93·4 %), followed by Xinjiang (26·89 (sd 6·50) ng/ml, 87·0 %). The lowest was found in Chongqing (22·19 (sd 8·70) ng/ml, 52·0 %) (Fig. 2(a)). There are significant differences in 25(OH)D concentration in some regions (Fig. 2(b)). The VitD nutritional statuses of children aged 2–6 years in various regions were significantly different (Table 5).

### Vitamin D nutritional status in children with different dietary habits

The surveyed children ate relatively high amounts of milk, eggs and meat products. Different dietary habits had an impact on the serum 25(OH)D concentration and the VitDD rate (Table 2). The serum 25(OH)D concentration and VitD sufficiency rate of children with dairy intake ≥ 3 times/week, egg intake ≥ 3 times/week and aquatic product intake ≥ 3 times/week were all relatively high (24·22 (sd 8·42) ng/ml, 66·1 %; 23·98 (sd 8·48) ng/ml, 65·7 %; 25·80 (sd 8·54) ng/ml, 74·1 %) (*P* < 0·05).

**Table 2:** 25(OH)D concentrations and VitD nutritional status of children with different dietary habits

				VitD nutritional status, *n* (%)					
		25(OH)D, ng/ml		Sufficient		Insufficient		Deficient	
Diary intake	*n*	Mean	sd	*n*	%	*n*	%	*n*	%
Dairy products intake									
< 3 times/week	507	20·88	7·25	255	50·3	209	41·2	43	8·5
≥ 3 times/week	1326	24·22	8·42	877	66·1	386	29·1	63	4·75
*P* value		< 0·000		< 0·000					
Eggs									
< 3 times/week	665	22·10	7·67	365	54·9	256	38·5	44	6·6
≥ 3 times/week	1168	23·98	8·48	767	65·7	339	29·0	62	5·3
* P* value		< 0·000		< 0·000					
Aquatic product intake									
< 3 times/week	1547	22·83	8·11	920	59·5	530	34·3	97	6·3
≥ 3 times/week	286	25·80	8·54	212	74·1	65	22·7	9	3·2
* P* value		< 0·000		< 0·000					
Meat intake									
< 3 times/week	516	22·65	8·26	306	59·3	178	34·5	32	6·2
≥ 3 times/week	1317	23·55	8·23	826	62·7	417	31·7	74	5·6
* P* value		0·036		< 0·400					

25(OH)D, 25-hydroxyvitamin D; VitD, vitamin D.

### Vitamin D supplementation and nutritional status

The overall VitD supplementation rate of the surveyed population was only 5·4 %. Before the age of 5 years, the VitD supplementation rate gradually decreased with age (Table 3). There was no significant difference in the VitD supplementation rate between the 4-year-old (3·3 %) and the 5–6-year-old groups (3·4 %). The serum 25(OH)D concentration in children with VitD supplementation was significantly higher than that in the non-supplemented population (29·37 (sd 6·80) ng/ml *v*. 23·15 (sd 8·16) ng/ml). Regardless of the dose, VitDD did not occur in the population receiving VitD supplementation (0·0 %, Table 3).

**Table 3:** VitD supplementation and nutritional status (*n* 1919)

VitD supplementation in the last 3 months			Age (years)								25(OH)D (ng/ml)		VitD nutritional status, *n* (%)					
	Total		2-		3-		4-		5–6		Mean	sd	Sufficiency		Inadequacy		Deficiency	
Supplemented	104	5·4	35	11·3	30	6·7	19	3·3	20	3·4	23·97	6·80	99	95·2	5	4·8	0	0·0
Non-supplemented	1815	94·6	275	88·7	418	93·3	560	96·7	562	96·6	23·15	8·16	1110	61·2	598	33·0	107	5·9
*P* value	< 0·000		< 0·000								< 0·000		< 0·000					

VitD, vitamin D.

### Geographical-environmental factors and vitamin D nutritional status at the sampling sites

**Fig. 3:**
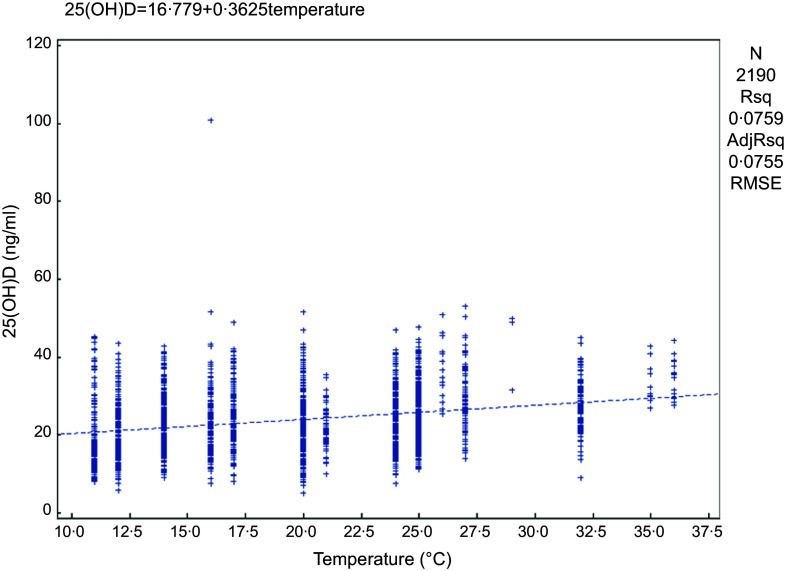
Correlation analysis of monthly mean temperature and serum 25(OH)D concentration at the time of sampling. 25(OH)D, 25-hydroxyvitamin D.

**Fig. 4:**
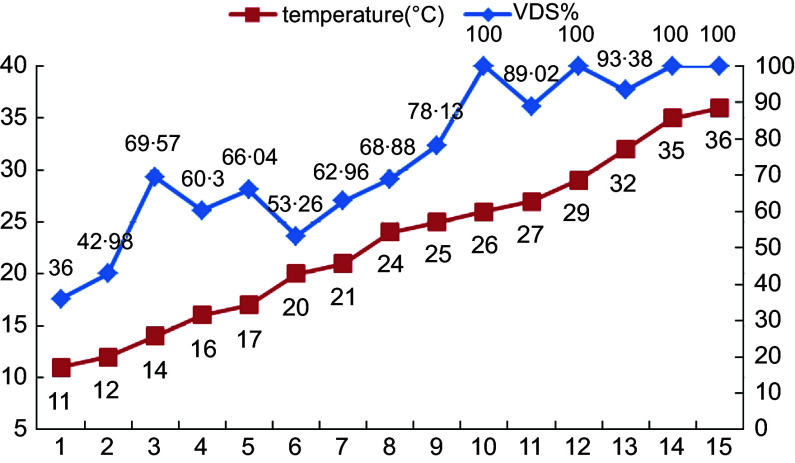
Monthly mean temperature at the time of sampling and the rate of VitD sufficiency. VitD, vitamin D.

Linear regression was used to analyse the effect of the latitude of the sampling sites and the monthly average temperature on VitD status in the population. There was a significant correlation between the monthly mean temperature and the serum 25(OH)D concentration in the population (25(OH)D = 16·779 + 0·3625 × temperature) (*P* < 0·05) (Fig. 3). The serum 25(OH)D concentration increased by 0·3625 ng/ml for every 1°C increase in temperature. From the broken-line graph of temperature and VitDS%, we can also see that at temperatures above 20°C, the proportion of VitD sufficiency gradually increased with increasing sampling temperature (Fig. 4); when the temperature was > 26°C, the proportion of VitD sufficiency often reached 100 %.

There was no correlation between the latitude of the sampling site and the serum 25(OH)D concentration or the prevalence of VitDD (*P* > 0·05).

### Risk factors for vitamin D deficiency and insufficient in preschool children

After adjusting for WAZ, HAZ and BMZ, we included sex, age, season, VitD intake, milk intake, egg intake, fish intake, latitude and temperature (monthly average temperature at the time of sampling) into the logistic regression analysis, and VitD status was the dependent variable (1 = VitD inadequacy: VitDI + VitDD; 0 = VitDS). The final correction factors included in the logistic regression model were WAZ, HAZ and BMZ. The results are shown in Tables 4a and 4b. Males have decreased odds of VitD inadequacy compared to females (OR, OR = 0·68, 95 % CI 0·52, 0·90). The odds of VitD inadequacy increase by 1·22 times for every year of age increase (OR = 2·22, 95 % CI 1·86, 2·64). Compared to winter, the odds of VitD inadequacy increase in spring (OR = 1·35, 95 % CI 0·91, 2·01) and decrease in summer (OR = 0·25, 95 % CI 0·09, 0·68) and autumn (OR = 0·26, 95 % CI 0·09, 0·74). Children who consume VitD supplements (OR = 0·08, 95 % CI 0·03, 0·28) and dairy (OR = 0·86, 95 % CI 0·78, 0·96) and egg products (OR = 0·83, 95 % CI 0·74, 0·93) more frequently have decreased odds of VitD inadequacy. When the monthly mean temperature at the sampling point varies between 11°C and 36°C, the odds of VitD inadequacy decrease by 11 % for every 1°C increase in temperature (OR = 0·89, 95 % CI 0·86, 0·93). All the above differences were statistically significant (*P* < 0·05) except for those associated with autumn. VitD status was not significantly correlated with household income or latitude (*P* > 0·05).

**Table 4a:** The factors affecting VitDI and VitDD in children aged 2–6 (*n* 1447)

Parameter	B	se	Wald *χ* ^2^	Unadjusted		*P*	Adjusted		*P*
				OR	95 % CI		OR	95 % CI	
Male	−0·38	0·14	1·96	0·67	0·54, 0·84	< 0·000	0·68	0·52, 0·90	0·006
Age	0·80	0·09	7·60	1·90	1·65, 2·19	< 0·000	2·22	1·86, 2·64	< 0·0001
Season (spring)	0·91	0·18	79·97	0·51	0·40, 0·66	< 0·000	1·35	0·91, 2·01	< 0·0001
Season (summer)	−0·77	0·36	25·81	0·05	0·02, 0·10	< 0·000	0·25	0·09, 0·68	0·033
Season (autumn)	−0·75	0·40	4·53	0·19	0·08, 0·43	0·333	0·26	0·09, 0·74	0·062
VitD intake	−2·48	0·61	3·49	0·07	0·02, 0·23	< 0·000	0·08	0·03, 0·28	< 0·0001
Milk intake	−0·15	0·05	16·56	0·76	0·68, 0·82	< 0·000	0·86	0·78, 0·96	0·005
Egg intake	−0·19	0·06	7·98	0·80	0·73, 0·88	< 0·000	0·83	0·74, 0·93	0·001
Temperature	−0·12	0·02	10·93	0·91	0·89, 0·93	< 0·000	0·89	0·86, 0·93	< 0·0001

VitDI insufficiency; VitDD, VitD deficiency; VitD, vitamin D.

Variables entered into the m odel: sex, age, season, vitD intake, milk intake, egg intake, fish intake, latitude and temperaure (monthly average temperature at the time of sampling).

Covariate: weight-for-age Z-score (WAZ), height-for-age Z-score (HAZ), and BMI Z-score (BMZ). VitD status was the dependent variable (1 = Vitamin D inadequate: VitDI + VitDD; 0 = VitDS).

Independent variable: Sex (0 = female, 1 = male), Season (1 = spring, 2 = summer, 3 = autumn, 4 = winter), VitD intake (0 = without vitD supplementation, 1 = with vitD supplementation), Milk intake and egg intake (9 = eat every day, 8 = 3–5 times/week, 7 = 1–2 times/week, 6 = little, 5 = never), Temperature is a continuous variable from 11°C to 36°C.

**Table 4b:** The factors affecting VitDI and VitDD in children aged 2–6 (*n* 1447)

				Unadjusted			Adjusted		*P*
Parameter	B	se	Wald *χ*^2^	OR	95 % CI	*P*	OR	95 % CI	
Sex									
Female	Reference								0·006
Male	−0·38	0·14	1·96	0·67	0·54, 0·84	< 0·000	0·68	0·52, 0·90	
Age	0·8	0·09	7·6	1·9	1·65, 2·19	< 0·000	2·22	1·86, 2·64	< 0·0001
Season									
Spring	0·91	0·18	79·97	0·51	0·40, 0·66	< 0·000	1·35	0·91, 2·01	< 0·0001
Summer	−0·77	0·36	25·81	0·05	0·02, 0·10	< 0·000	0·25	0·09, 0·68	0·033
Autumn	−0·75	0·4	4·53	0·19	0·08, 0·43	0·333	0·26	0·09, 0·74	0·062
Winter	Reference								
VitD intake									
Without VitD supplementation	Reference								
With VitD supplementation	−2·48	0·61	3·49	0·07	0·02, 0·23	< 0·000	0·08	0·03, 0·28	< 0·0001
Milk intake									
Never drink milk	Reference								
Milk intake	−0·15	0·05	16·56	0·76	0·68, 0·82	< 0·000	0·86	0·78, 0·96	0·005
Egg intake									
Never eat egg	Reference								
Egg intake	−0·19	0·06	7·98	0·8	0·73, 0·88	< 0·000	0·83	0·74, 0·93	0·001
Temperature	−0·12	0·02	10·93	0·91	0·89, 0·93	< 0·000	0·89	0·86, 0·93	< 0·0001

VitDI insufficiency; VitDD, VitD deficiency; VitD, vitamin D.

Variables entered into the model: sex, age, season, VitD intake, milk intake, egg intake, fish intake, latitude and temperature (monthly average temperature at the time of sampling).

Covariates: weight-for-age Z-score (WAZ), height-for-age Z-score (HAZ), and BMI Z-score (BMZ). VitD status was the dependent variable (1 = Vitamin D inadequacy: VitDI + VitDD; 0 = VitDS).

Independent variable: Sex (0 = female, 1 = male), Season (1 = spring, 2 = summer, 3 = autumn, 4 = winter), VitD intake (0 = without VitD supplementation, 1 = with VitD supplementation), Milk intake and egg intake (9 = eat every day, 8 = 3–5 times/week, 7 = 1–2 times/week, 6 = little, 5 = never), Temperature is a continuous variable from 11°C to 36°C.

**Table 5:** VitD nutritional status of children in different regions

Regions		VitD nutritional status, *n* (%)					
	*n*	Deficient		Insufficient		Sufficient	
Fujian	181	1	0·6	11	6·1	169	93·4
Guizhou	198	9	4·6	70	35·4	119	60·1
Qinghai	151	7	4·6	60	39·7	84	55·6
Chongqing	827	79	9·6	318	38·5	430	52·0
Sichuan	210	1	0·5	47	22·4	162	77·1
Xinjiang	200	3	1·5	23	11·5	174	87·0
Yunnan	201	3	1·5	47	23·4	151	75·1
Shanxi	224	11	4·9	73	32·6	140	62·5
*P* value		< 0·0001					

VitD, vitamin D.

## Discussion

VitD includes VitD2 and VitD3^(23)^. VitD2 is most common in plant food sources such as mushrooms and ergot and in fortified foods^(24,25)^. During exposure to sunlight, 7-dehydrocholesterol in the skin is converted to VitD3^(26)^. In recent years, with the development of China’s economy, the proportion of urbanisation has further expanded, and people’s living habits have undergone major changes. Their diet is richer, and more attention is given to nutrient supplementation. At the same time, children enter kindergarten early, resulting in a significant reduction in outdoor activities. In recent years, there have been few large-scale population surveys of VitD status in China, especially of preschool children. Because of the difficulty of sampling, since the 2010–2013 national survey, there has been no multicentre VitD status survey of preschool children. The VitD nutritional status data of Chinese preschool children in the past 5 years were generated from outpatients^(3,4,14–16)^.

This study used national large-scale multicentre data to analyse the epidemiological characteristics and influencing factors of the VitD nutritional status of children aged 2–6 years in China. The results showed that the rates of VitDD and insufficiency reached 5·2 % and 29·6 %, respectively, which were lower than the national multicentre data in 2013^(12)^, indicating that the overall VitD nutritional status has improved. Blood collection season, dietary habits, VitD supplementation and sampling temperature were correlated with VitD nutritional status.

Season is an important factor affecting 25(OH)D concentrations. Like previous studies, this study suggests that in almost all age groups, the 25(OH)D concentration is the lowest in winter and spring and highest in summer^(3,7,8,27–29)^. The intrinsic reason for the difference in seasonal 25(OH)D concentration is the intensity of seasonal sunlight exposure, the outdoor activities of the population and the surface area of the skin exposed to sunlight. In summer, the UV intensity is relatively high, more of the skin is exposed to sun and so the skin makes relatively high amounts of VitD.

With increasing age, the serum 25(OH)D concentration gradually decreased, and the VitDD rate gradually increased, which is consistent with the data obtained by the healthcare departments of various hospitals in recent years^(14–16)^. This may be related to the decrease in the VitD supplementation rate. The VitD supplementation rate of children after 2 years of age was generally low (5·4 %), and the supplementation rate further decreased with increasing age. With increasing age, children’s outdoor activities gradually decrease^(17)^. When VitD is gradually consumed by the body, the serum 25(OH)D concentration gradually decreases. This study also found that no VitDD occurred in the population who received VitD supplementation. We did not have any data on the amount of outdoor activities. More regional and population census data are needed, especially of preschool children.

The serum 25(OH)D concentrations and VitD sufficiency rates of boys were higher than those of girls, in line with previous studies^(14,17,30)^. Previous studies have found a sex difference in VitD, which may be because boys have less body fat than girls^(17)^. However, in this study, there was no sex difference in the body fat distribution of preschool children, which may be because the amount of outdoor activities in boys is greater than that of girls, and girls pay more attention to sun protection when they go out. The relationship between sex and VitD is relatively complex, there are few studies on this aspect and further studies are needed.

Previous studies suggest that in high-latitude areas, especially north of 35°N, the UV intensity is relatively low in winter and spring, and the skin makes little VitD^(5–7,9)^. In this survey, after adjusting the sampling season, age, sex and other indicators, latitude did not have a significant effect on the VitD nutritional status, and there was no significant difference in the 25(OH)D concentration between the northern and southern regions. A cross-sectional survey conducted at a hospital in Taiyuan, China, also found that latitude had no significant effect on VitD nutritional status^(13)^. China is a large country with relatively large differences in topography and culture. Even in the same latitude area, there was still a large difference in VitD status between cities, such as between Guizhou, Yunnan and Fujian, in this study. The sampling latitudes of Yunnan and Fujian were relatively close, and the annual average sunshine duration in Yunnan is longer than that in Fujian, but the serum 25(OH)D concentration in Fujian was significantly higher, which may be related to their higher consumption of fish. In this study, 21·55 % of children in Fujian (Fuzhou) ate fish daily, while only 4·48 % ate fish daily in Yunnan (Kunming). The annual average temperature in Fuzhou is higher than that in Kunming, and the skin exposure time is longer than that in Kunming, which may also be the reason for the difference. In the high-latitude regions of this survey, the VitDD rates in Qinghai (36·98°N) and Guizhou (26·58°N) were the same, and the serum 25(OH)D concentrations were similar. The 25(OH)D concentration in Chongqing was the lowest in the low-latitude regions, and the 25(OH)D concentration was the highest in Xinjiang (47·87°N), which completely contrasts with the previous results. With changes in human lifestyle, environmental pollution has been aggravated, and the amount of solar UVB that reaches the surface has decreased due to air pollution^(31)^. Preschool children of all latitudes spend less time outdoors, so the influence of latitude is greatly reduced. Although Xinjiang is located in a high-latitude region, the sampled children were all Uyghurs, with lighter skin than Han Chinese. In addition, the annual average sunshine duration in Xinjiang is relatively long, the population eats large amount of dairy products and the sampling time was spring, so the 25(OH)D concentration was relatively high. Different studies have all found that the 25(OH)D concentration in Chongqing is relatively low^(12,17)^, which may be because the weather in Chongqing is humid and foggy, the surrounding mountains are relatively high and the sunshine duration at the sampling site is relatively short.

Previous studies have suggested that food sources of VitD are relatively small and have little effect on human VitD. Even so, this study found that the intake of dairy products and egg products had a significant impact on the VitD nutritional status. This may be because when there is less sunlight exposure and VitD supplementation, the relative influence of food on the nutritional status of human VitD increases. In this study, the proportion of aquatic product intake was relatively small, and only 15·6 % of the population ate aquatic products more than three times per week. Many inland areas were sampled, so freshwater fish and shrimp made up a greater proportion of the food intake. This may be one reason why aquatic products had no significant effect on VitD nutritional status in the logistic regression analysis.

Although latitude had little effect on 25(OH)D concentrations, the sampling area had a significant impact on the VitD nutritional status. Samples will be affected not only by the latitude and temperature of the sampling site but also by the economic and medical development status of the area and its health policy. The intake of VitD preparations had the greatest impact on VitD status (OR = 10·64). The monthly average temperature at the time of sampling showed a linear correlation with the serum 25(OH)D concentration. When the temperature was lower than 20°C, the relationship between air temperature and the percentage of VitD sufficiency (VitDS%) was not clear. This may be because the degree of skin exposure was lower when the temperature was too low, and the effect of sunlight on VitD nutrition was weaker. After the temperature reached higher than 25°C, only one person had VitDD. This study also investigated the effect of household income per capita on VitD nutritional status and found that after correction for many factors, household income per capita had no significant effect on the 25(OH)D concentrations of children aged 2–6 years.

With the development of Chinese society and changes in its environment and lifestyle, the factors affecting the VitD nutritional status have changed. Some primary care physicians still follow the guidelines of the Paediatrics Branch of the Chinese Medical Association and recommend that all children receive no less than 400 IU/d of VitD only from 2 weeks to 2 years after birth^(32)^ and stop taking VitD supplements at the age of 2 years. At the same time, the Chinese public has doubts about long-term VitD supplementation^(13)^ and believes that benefit of VitD is only to promote Ca absorption. Further strengthening the education of VitD supplementation, recommending regular monitoring of the 25(OH)D concentration in children and improving the awareness of the importance of VitD supplementation level among healthcare personnel and family members should all be addressed in our health efforts and in the development of health policies. The National Health Organization can tell doctors and parents which regions and populations are most likely to experience VitDD and need to strengthen supplementation. In China, many parents do not want to constantly provide their children with VitD supplementation. They are afraid of VitD overdose. In this study, VitDD rates were extremely low among preschool children in summer (0·4 %) and coastal cities (0·6 %). For these preschool children, VitD supplementation can be intermittent based on their level of exposure to sunlight during the summer in order to increase the long-term compliance with VitD supplementation.

### Conclusion

VitDD in children aged 2–6 years is still prevalent in China. Although it is better than it was in 2010–2013, it is still an important public health problem. With changes in lifestyle, the influencing factors of VitD nutritional status have also changed. In China, latitude is not the main factor affecting the 25(OH)D concentration of preschool children; rather, the temperature at the time of sampling, the intake of eggs and dairy products, the sampling season, and the sampling area have more significant effects on the VitD nutritional status of children aged 2–6 years. It is necessary to further strengthen the education and policy development of VitD supplementation to improve the overall VitD nutritional status of Chinese children.
